# Temperate UV-Accelerated Weathering Cycle Combined with HT-GPC Analysis and Drop Point Testing for Determining the Environmental Instability of Polyethylene Films

**DOI:** 10.3390/polym13142373

**Published:** 2021-07-20

**Authors:** Celine Moreira, Richard Lloyd, Gavin Hill, Florence Huynh, Ana Trufasila, Faith Ly, Hasan Sawal, Christopher Wallis

**Affiliations:** Polymateria Limited, i-Hub, Imperial College White City Campus, 84 Wood Lane, London W12 0BZ, UK; cm@polymateria.com (C.M.); rl@polymateria.com (R.L.); gh@polymateria.com (G.H.); fh@polymateria.com (F.H.); at@polymateria.com (A.T.); fl@polymateria.com (F.L.); hs@polymateria.com (H.S.)

**Keywords:** UV-accelerated weathering, polyethylene, outdoor exposure, weathering correlation, HT-GPC, carbonyl index, molecular weight

## Abstract

Polyethylene films are one of the most frequently used packaging materials in our society, due to their combination of strength and flexibility. An unintended consequence of this high use has been the ever-increasing accumulation of polyethylene films in the natural environment. Previous attempts to understand their deterioration have either focused on their durability using polymer analysis; or they have focused on changes occurring during outdoor exposure. Herein, this study combines those strategies into one, by studying the chemical and physical changes in the polyethylene structure in a laboratory using molecular weight and IR spectroscopic mapping analysis, combined with temperate UV-accelerated weathering cycles. This approach has been correlated to real-world outdoor exposure timeframes by parallel testing of the sample polyethylene films in Florida and France. The formation of polyethylene microparticles or polyethylene waxes is elucidated through comparison of drop point testing and molecular weight analysis.

## 1. Introduction

Polyethylene is the most ubiquitous polyolefin plastic resin globally. Its production makes up 28% of the total global production of plastics, estimated at 100 million tons in 2018 [1]. The reason for its domination of the market is two-fold: the first is its price. At (approximately) 0.59 euros per kilo, it is one of, if not the cheapest, plastic resin available on the market [2]. The second reason is its versality. Within its three most common forms of linear low density (LLD), low density (LD) and high density (HD), polyethylene materials can be tailored with a large number of stand-alone or combined properties [3,4]. For example, a HDPE container creates a non-reactive sturdy receptacle, whereas combining LLDPE and LDPE in a film produces packaging that is both flexible and strong. The versatile utility of polyethylene, particularly in films, has consequently led to its regular use in film packaging, agriculture mulch films and film linings for composite paper products, such as coffee cups [3,4]. The unintended consequence of this ubiquitous use for the last 30 years has been the accumulation of polyethylene-based materials in the natural environment [5,6,7,8,9]. A combined effect of lack of investment in infrastructure and waste management systems, as well as illegal dumping of plastic waste on an industrial scale, has led to certain areas of the globe becoming sources of fugitive plastic pollution, particularly polyethylene [5,6,7]. As an example, a recent survey commissioned by the Ocean Conservancy found 4.7 million food wrappers in beach clean-up projects, the vast majority of which would have been made from polyethylene. In 2020, for the first time in 30 years, these products had ‘dethroned’ cigarette butts as the largest proportion of fugitive plastics in the natural environment [10].

Therefore, in this context, there is a pressing need to add to our understanding of the role of weathering on how fugitive polyethylene films interact with the natural environment [11]. Better knowledge is needed of the physical and chemical changes that occur when these materials are stimulated by the interaction of the polyethylene chains with photons, oxygen and water molecules [12]. A great deal more research has been conducted into stabilizing polyethylene by introducing anti-oxidants or light stabilizers than in investigating their inevitable breakdown due to environmental exposure [13,14]. Although not present in all commercial resins, the majority of HDPE and LLDPE resins contain anti-oxidants to help stabilize their use in manufacturing processes. These additives can be responsible for time delays before natural degradation pathways can occur as additive levels vary [15]. There is a need to better understand the natural degradation process, the effect additives can have on the degradation pathways, as well as more advanced laboratory simulation to aid in the elucidation of Service Lifetime Predictions (SLP) [11,12].

These lifetime predictions are critical when the polyethylene or polypropylene plastics are used as the material in a commercial product or application. One example recently studied was the SLP of polymers used as high-voltage insulators on overhead electric power cables for trains in India [16]. More specifically to polyolefins, studies have been conducted that have shown the correlation between the durability of polypropylene in laboratory accelerated weathering compared to outdoor exposure in different regions of China [17] and Japan [18]. The correlation between the accelerated weathering of polyolefins in a laboratory and real-world outdoor exposure is critical to validate the performance of the polyolefin when used in a real-world application. International standards currently govern the use of UV-accelerated weathering, namely ASTM D4329-13 [19] and ISO 4892-3 [20]. Moreover, standards governing the outdoor exposure methodologies for plastics, such as ASTM G113 [21], state that indoor accelerated tests should match results obtained through outdoor exposure.

Likewise, any technologies designed to increase the rate of chemical transformations occurring within the polyethylene chains need to be evaluated by simulated laboratory techniques of relevancy to real-world exposure conditions and timeframes. To that end, we recently published, a novel temperate UV-accelerated weathering cycle for evaluating polyethylene materials, via a laboratory technique that has been correlated to outdoor exposure conditions [22]. This relative correlation is important in assessing the utility of the Service Life Prediction of materials, to which a defined point of failure is equally critical to define in the analysis [11]. This work is also important as it provides the evidence base for incorporating new laboratory weathering cycles into national or international standards. This follows the recent example published by Kunioka et al., in which the weathering cycles were ring tested at various sites in Japan, building the evidence base for standardized testing methods at both the national level of Japan, as well as at the international level of ISO standardization committees [18]. For polyethylene samples, high-temperature gel permeation chromatography was found to be an excellent and underused polymer analytical technique to demonstrate quantifiably the changes in the length and structure of the polymeric chains within the polyethylene matrix. Combining this methodology with the measurement of the carbonyl index allowed for the evaluation of the point of failure of the polyethylene samples. In the case of the standard polyethylene, the determination of the deterioration of the film was elucidated, resulting in the formation of secondary polyethylene microparticles. In contrast, the same polyethylene sample containing Polymateria’s biotransformation technology was tested alongside this. The chemical changes caused the polyethylene chain structure to significantly decrease to polyethylene waxes containing a high concentration of carbonyl groups, a result in stark difference to the standard polyethylene sample [22].

With ever-increasing scrutiny of the interactions of fugitive polyethylene plastics with the elements in the natural environment, better laboratory techniques for simulating the deterioration of polyethylene are the key missing link in combining the theoretical studies with the collection of samples from the environment—moreover, combining the environmental element with ‘classical’ polymer analysis designed for assessing the chemical and physical changes within the polyethylene matrix, notably molecular weight analysis and infrared spectroscopy. The objective in this research is to define more precisely the timeframe of exposure to environmental stimuli that polyethylene films could withstand before deterioration, either physical or chemical, would occur. In addition, these techniques will be used to demonstrate their utility in evaluating technologies within polyethylene designed to shorten their lifetime and reduce their fugitive impact. Quantitative analysis of the rate of change in the polyethylene chains will help to identify the kinetics and the polymer composition. The overall intention is to experimentally elucidate the theoretical calculations [9], and/or environmental observations [10] previously made with respect to polyethylene deterioration induced by outdoor exposure.

## 2. Materials and Methods

### 2.1. Materials

The samples for the accelerated and outdoor weathering study were produced on a commercial extrusion line, where additive dosage can be well controlled. The standard stretch film (PE-01) (produced at Norner AS, Stathelle, Norway) is a green multi-layer polyethylene film (17 µm thick) composed of (by weight) 52% LLDPE (Versalis CL10 (ENI, Rome, Italy)), 40% mLLDPE (Daelim XP9400 (Daelim Industrial Co., Seoul, South Korea)), 5% VLLDPE (Versalis CLD0 (ENI, Rome, Italy)) and 3% Green MB (Ampacet 1701273-E). The films containing the biotransformation technology had the same polymer resin composition with the addition of the drop-in additive dosed at 2% by weight (17 µm thick). (To compensate for the Masterbatch addition, the LLDPE (Versalis CL10 (ENI, Rome, Italy)) proportion was reduced to 50% by weight. The thickness of all films was kept constant at 17 µm. (See Table 1 for sample description.)

### 2.2. Temperate Accelerated Laboratory Weathering

The polyethylene (PE) films testing was performed using a QUV-A fluorescence tester from Q-Lab (Q-Lab, Homestead, FL, USA) where irradiance and black panel temperature are calibrated at 340 nm. The irradiance setpoint was 0.8 W/m^2^ and the black panel temperature (BPT) was 60 °C for 1 h with 23 h dark at 60 °C in 24 h repeated over 14 days. The methodology of rotation of the samples during the testing period was performed in accordance with ISO 4892-3:2016 [20].

### 2.3. Outdoor Exposure of Films

Outdoor weathering was carried out at the Q-Lab Weathering Research Service (Q-Lab, Homestead, FL, USA) testing site close to Miami, Florida, following the specification of ASTM D1435-20 [23] and the Atlas Material Testing Technology B.V testing site in Sanary Sur Mer, France; and the sampling, recording and analysis were performed in accordance with the specification of ISO/IEC 17025:2017 [24]. Samples were cut to 30 × 15 cm and held in a HDPE net with a 1 mm mesh to prevent sample cross-contamination and loss due to wind during the aging process. The samples were mounted at 45°, south facing, and weathering data were recorded in the vicinity of the test racks for every day of testing. Average daily temperature, humidity, rainfall and irradiance (UV irradiance measured between 285 and 385 nm with Eppley TUVR 295–385 nm).

### 2.4. Infrared Analysis and FTIR Spectroscopic Mapping

FTIR spectroscopic mapping was carried out on either a Thermofisher Scientific (Waltham, MA, USA) Nicolet iS10 or a Nicolet iS5 equipped iD7 Diamond ATR between 4000 and 600 cm^−1^ at a resolution of 4 cm^−1^. The total carbonyl index was calculated using the SAUB method [25], where CI = Area_(1850–1650)_/Area_(1500–1420)_. This method is recommended as it accounts for the total concentration of carbonyl species generated during the weathering.

FTIR microscopy was carried out on a Thermofisher Scientific (Waltham, MA, USA) iN10 IR Microscope with Dual Detector operating under reflectance. Bulk film mapping was carried out by placing the samples in good contact with a gold-coated microscope slide and optimizing the reflected IR signal. A total of 16 scans at 4 cm^−1^ resolution between 4000 and 400 cm^−1^ were carried out at each point (20 microns apart). The maps produced were then normalized to the baseline and converted to a carbonyl index map using the Omnic Picta software, using the above relationship of CI = Area_(1850–1650)_/Area_(1500–1420)_. As well as the resulting 2D maps, the software also produces a 3-D tomograph indicating areas of higher and lower intensity.

### 2.5. Molecular Weight Analysis

Molecular weights of the samples were carried out using an Agilent 1260 Infinity II High Temperature Gel Permeation Chromatography system equipped with Refractive Index detector, 2× Olexis PL-Gel columns (Agilent, Santa Clara, CA, USA) and calibrated using PS standards operating in 1,2,4-trichlorobenzene (TCB). Samples were measured at 160 °C at a flow rate of 1 mL/min and samples were allowed to dissolve in TCB for 4 h and analyzed according to ASTM D6474-20 [26]. For most HT-GPC experiments, only a single measurement was recorded. This, is due to the time and cost involved in running samples and because the HT-GPC has high reproducibility when samples are prepared correctly.

### 2.6. Drop Point Test

Drop point testing was performed using best practice in accordance with ASTM D127-19 [27] and according to a method described in a thesis titled Wax Characterization by Instrumental Analysis [28]. The protocol requires pre-melting of the resulting wax and adding the hot melt to the end of a high-temperature glass thermometer. The thermometer is suspended in a closed glass vessel immersed in a glycerol heating bath and heated at a rate of approx. 0.5 °C/min. The temperature at which a droplet of molten material drops from the wax was recorded. Each drop point test is reported as an average of a triplicate (Table S5). The methodology stipulates that to be considered a wax, the drop point must be less than 140 °C. It is further noted that thermoplastic samples do not drop from the thermometer due to their high melt viscosity under this temperature. If the sample recorded no dropping point at 140 °C, the sample was deemed to have failed and the testing was stopped.

## 3. Results

### 3.1. Outdoor Exposure Sites

The weathering of the polyethylene (PE) samples was carried out at the Q-Labs test site in Florida (US)), which is considered a tropical savanna climate (Aw) test environment, and Sanary Sur Mer (France), which is considered a Mediterranean climate (Csa) according to the Koppen climate classification [29]. The samples were angled 45 °C to the sun on racks. The average daily temperature and UV irradiance were recorded (285 and 385 nm) over the total calendar period of 4 months of the experiment (Figure S1). The PE samples were exposed outdoors for a total of 90 days for Florida during the months of June to August and the months of August to October. This reflected the weathering conditions experienced during both the wetter and drier times of the year. The films in France were exposed for a total of 120 days during the months of September to December. This reflects both the hotter (summer) period of the year, as well as the cooler (winter) period of this geographical climate.

### 3.2. Weathering Data and Conditions for Exposure in France

**Figure 1:**
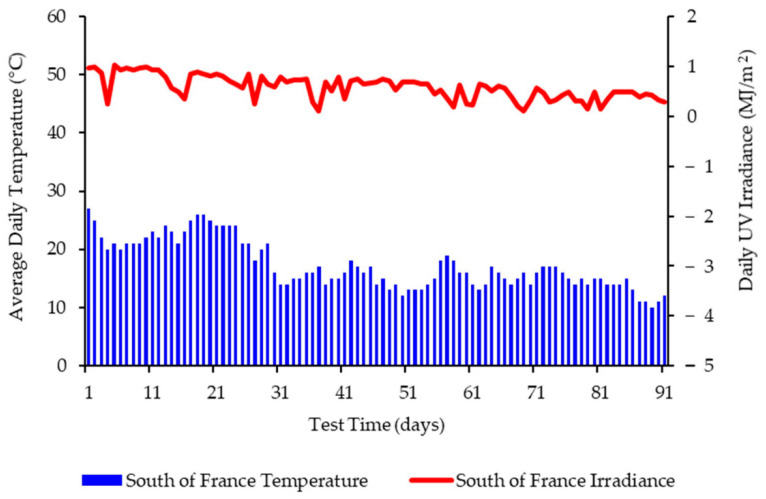
Weathering data for South of France (September–December 2020) showing daily average temperatures and daily irradiance values up to 91 days.

The specialist site chosen in France for these studies was equipped with weatherometers capable of capturing and recording the climatic conditions over the time of the exposure. Figure 1 presents the average daily temperature and UV irradiance experienced by the films at this site during the period of September–December. Average daily rainfall and average humidity were also recorded (see Figure S1).

### 3.3. Weathering Data and Conditions for Exposure in Florida

**Figure 2:**
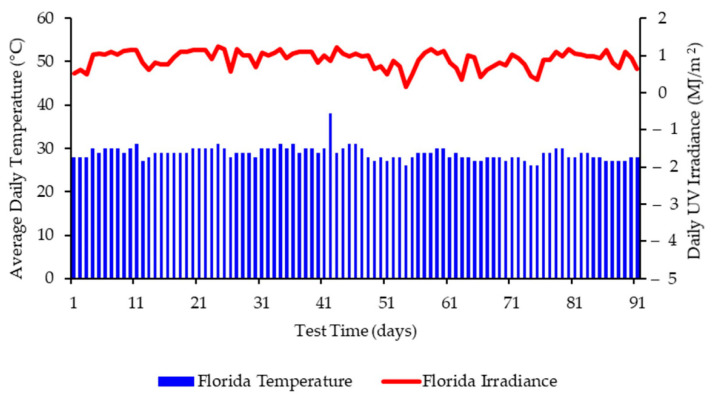
Weathering data for Florida (July to September 2020) showing daily average temperatures and daily irradiance values up to 91 days.

**Figure 3:**
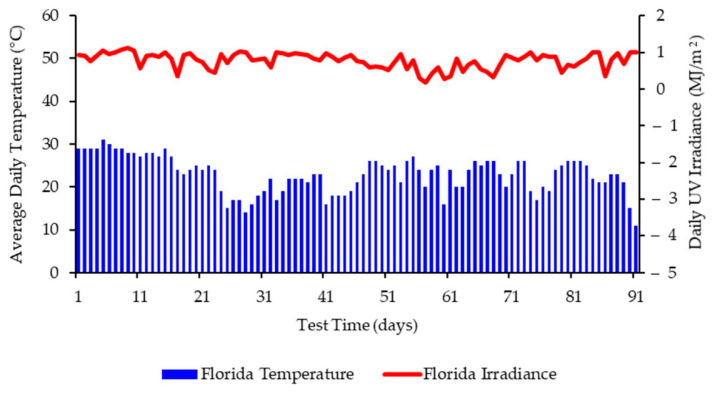
Weathering data for Florida (October 2019 and January 2020) showing daily average temperatures and daily irradiance values up to 91 days.

The specialist site chosen in Florida for these studies was equipped with weatherometers capable of capturing and recording the climatic conditions over the time of the exposure. Figure 2 presents the average daily temperature and UV irradiance experienced by the films at this site during the period of June–August, whereas Figure 3 presents the average daily temperature and UV irradiance experienced by the films at this site during the period of September–December. Average daily rainfall and average humidity were also recorded (see Figures S2 and S3).

### 3.4. Molecular Weight Changes during Temperate Laboratory Exposure and under Outdoor Exposure in France

**Figure 4:**
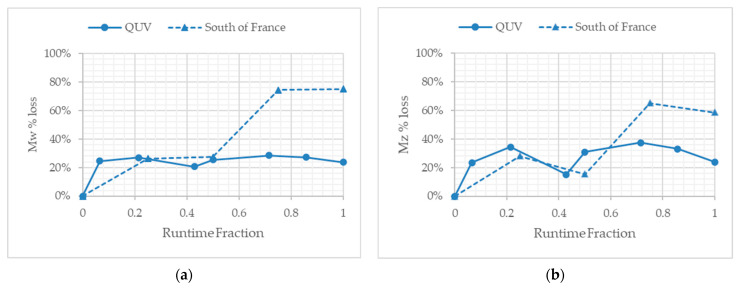
(**a**) The percentage loss of the weight-average molecular weight versus time for PE-01 and PE-03, under temperate UV-accelerated laboratory conditions (solid line) and outdoor exposure in France (dotted line), respectively. (**b**) The percentage loss of the higher average weight-average molecular weight versus time for PE-01 and PE-03, under temperate UV-accelerated laboratory conditions (solid line) and outdoor exposure in France (dotted line), respectively.

**Figure 5:**
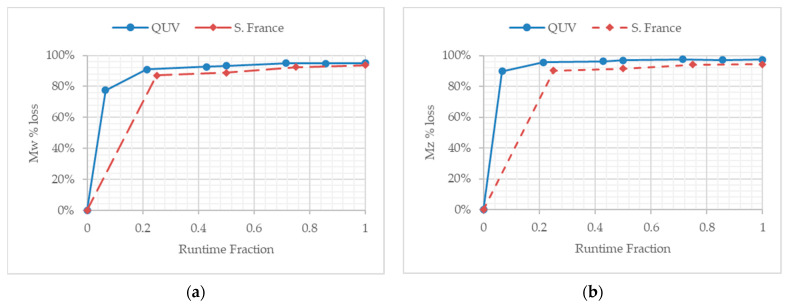
(**a**) The percentage loss of the weight-average molecular weight versus time for PE-02 and PE-04, under temperate UV-accelerated laboratory conditions (solid blue line) and outdoor exposure in France (dotted red line), respectively. (**b**) The percentage loss of the higher average weight-average molecular weight versus time for PE-02 and PE-04, under temperate UV-accelerated laboratory conditions (solid blue line) and outdoor exposure in France (dotted red line), respectively.

The molecular weight analysis was obtained by HT-GPC analysis using TCB as solvent. All the samples completely dissolved in the TCB, suggesting that no gel or insoluble formations were produced as a result of the QUV or outdoor weathering. As previously reported [22], the key changes in molecular weight are more clearly observed when the loss and percentage (%) loss of weight-average molecular weight (Mw) and higher weight-average molecular weight (Mz) are compared and plotted (Table 2 and Figure 4). Furthermore, the use of the runtime fraction [16] allowed for the comparison of temperate laboratory accelerated and outdoor exposure studies to be compared and contrasted (Figure 4 and Figure 5).

PE-01 showed a % loss in Mw and Mz at the end of the temperate laboratory accelerated weathering of 27% and 24%, respectively. PE-02, on the other hand, showed Mw and Mz% losses of 95% and 97%, respectively, after the same period of UV weathering. In the case of PE-01, the majority of the molecular weight losses appear to have occurred in the first 24 h (0.067 fraction of the total runtime), after which no significant further molecular weight losses are observed. By comparison, PE-02 loses the majority of the Mw and Mz values in the first 72 h. Whilst this loss takes longer than PE-01, the degree of loss is far greater, as PE-02 loses 91% and 96% of its Mw and Mz values, respectively. The difference does not appear to relate to the green pigment added to the films, as this remains unchanged in terms of the hue of the green color throughout the experiment. Thus, the difference is related to the biotransformation technology in PE-02. The loss in molecular weight observed for PE-01 is consistent with the known phenomenon of UV degradation of polyethylene [15]. This work, however, highlights in more detail the loss of either Mw or Mz needed to cause fragmentation of the macroplastic film into microparticles of polyethylene. The effect of the biotransformation technology will be described later on.

PE-03 showed a % loss in Mw and Mz at the end of the outdoor exposure in France of 67% and 59%, respectively. PE-04, on the other hand, showed Mw and Mz% losses of 94%, respectively, after the same period of climatic exposure. Similarly to the temperate laboratory UV-accelerated weathering, the initial molecular weight losses were seen in the first 30 days, which equals to a 0.25 fraction of the total runtime, compared to 0.21 for the laboratory samples (Table 3). The green hue of the films did not change over time. However, sample recovery of PE-04 became very difficult as physical erosion of the film alongside the chemical changes had clearly caused the severe loss of the physical properties of the sample. PE-03, on the other hand, showed some indication of physical erosion due to the climatic conditions, resulting in small fragments of the film sample, which had detached from the film over the exposure time (Figure 4).

The use of the runtime fraction allows for the comparative changes observed over time to be plotted (Figure 4). PE-01 and PE-03 show a similar loss in Mw and Mz of approximately 28% after 60 days of UV irradiation (0.5 runtime fraction). After 60 days, however, the outdoor exposed sample of PE-03 shows further losses in Mw and Mz, increasing to 67% and 59%, respectively, after the total time of UV exposure. We postulate that this secondary loss of molecular weight is due to physical erosion of the sample, which, outdoors, causes a greater surface area of the sample exposed to sunlight irradiation, and thus leads to further loss of the molecular weight structure of the polyethylene chains. Further studies will be conducted to evaluate this phenomenon. In contrast, no significant differences are observed between samples PE-02 and PE-04. The rate and degree of reduction in Mw and Mz appears to be similar under both types of UV and outdoor exposure, reaching an observed plateau by 0.3 fraction of the total runtime (Figure 5).

### 3.5. Molecular Weight Changes during Outdoor Exposure in Florida at Different Times of the Calendar Year

**Figure 6:**
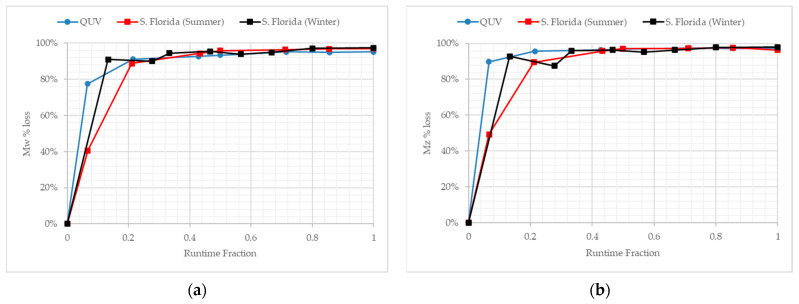
(**a**) The percentage loss of the weight-average molecular weight versus time for PE-02, PE-05 and PE-06, under temperate UV-accelerated laboratory conditions (solid blue line) and outdoor exposure in Florida summer (solid red line), and Florida winter (solid black line), respectively. (**b**) The percentage loss of the higher average weight-average molecular weight versus time for PE-02, PE-05 and PE-06, under temperate UV-accelerated laboratory conditions (solid blue line) and outdoor exposure in Florida summer (solid red line), and Florida winter (solid black line), respectively.

The PE samples were exposed in Florida at two distinct times of the year to represent the seasonal variables of that geographical climate. Both PE samples exhibited Mz losses of 96% after the first 30 days (Table 4). PE-05 reaches a Mw of 4694 Da and an Mz of 16,885 Da after the total exposure period (Figure 6). PE-06 reaches similar values of an Mw of 3936 Da and an Mz of 9179 Da after the total time of exposure despite the average temperature being 5.9 °C colder (Table S1).

### 3.6. IR Mapping Images of the Surfaces of the Films during Exposure

Following on from our previous work [22], we noted the value in using transmission or reflectance IR spectroscopic analysis for determining the chemical transformations across the surface and through the bulk of the sample. We also noted, however, that single-point analysis limited the ability to observe whether the changes were homogeneous. To resolve this, we have employed IR mapping as a tool to check whether oxidation is occurring homogeneously across the samples. By mapping the carbonyl index, it is possible to compare the degree of oxidation over exposure time and monitor whether the process occurs homogeneously or via “hotspot” areas of high/low activity. IR mapping is proving to be a useful tool that can be expanded from thin-film surfaces to cross-sectional areas for thicker samples.

IR microscopy uses a combination of optical microscope images and a focused IR laser to excite any covalent dipoles in the path of the beam, and the excitation spectrum is then either transmitted to the detector below the path or reflected back to the detector above the path [30,31,32]. In this way, the spectrum position can be correlated to the physical position on the sample. Resolution is controlled largely by increasing or decreasing the number of sampling points, though at <20 micron spacing, there will be some spectral overlap due to the wavelengths of the laser and the width of the beam. Thus, PE-01 was analyzed as a defined 1 mm by 1 mm square.

**Figure 7:**
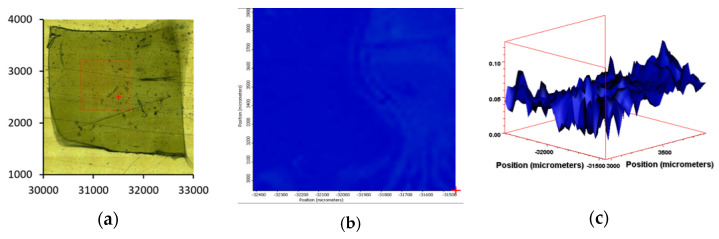
(**a**) Microscopic image of PE-01. The red square represents the area under mapping evaluation. (**b**) IR ‘hotspot’ CI map generated as a result of multiple IR spectra taken over the highlighted area. (**c**) Corresponding 3-D tomograph of the CI variations over the analyzed surface of PE-01.

Using the methodology detailed within, we then extended this analysis to the PE samples containing the biotransformation technology. Similarly to PE-01, the ATR-IR software was setup to represent the SAUB calculated CI as a ‘hotspot’ thermal mapping of the surface of the film sample (Figure 7). Additionally, the software calculated a 3-D graphic of the area analyzed, where the intensity of the CI can be represented across the surface of the film as a topological graphic indicating areas of higher and lower intensity. Encouragingly, the 3-D tomographs suggested that despite minor fluctuations, the entire surface analyzed produced a consistently elevated CI measurement.

**Figure 8:**
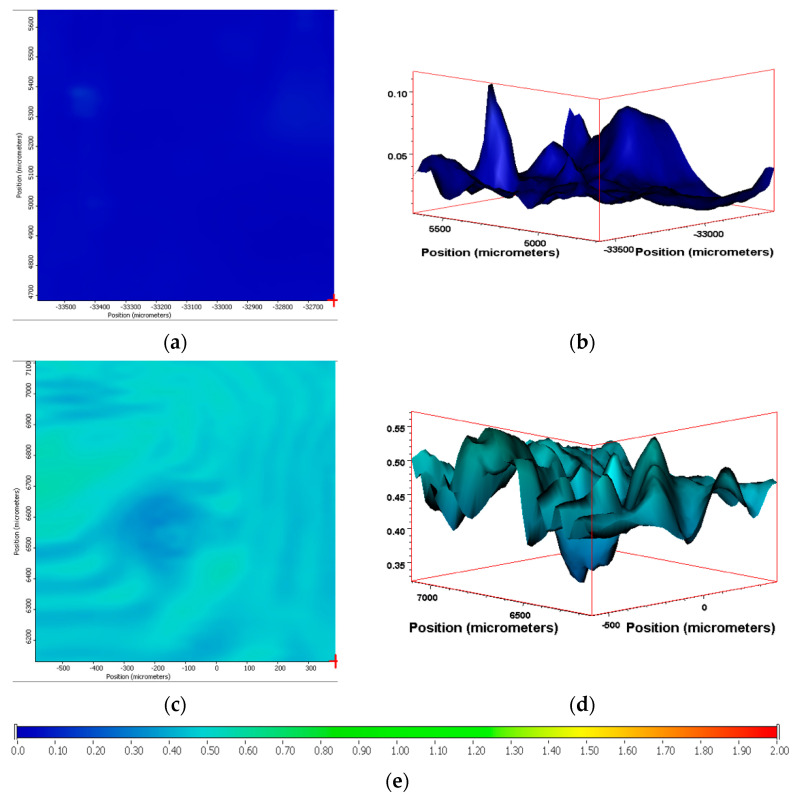
(**a**) IR ‘hotspot’ CI map and (**b**) corresponding 3-D tomograph of the CI of PE-01 after 14 days of temperate UV-accelerated weathering. (**c**) IR ‘hotspot’ CI map and (**d**) corresponding 3-D tomograph of the CI of PE-03 after 120 days of outdoor exposure in France. (**e**) Scale of ‘hotspot-map’ relative to CI values measured from IR spectra.

Sample PE-01, unsurprisingly, gave a very low CI throughout the surface area analyzed. Both the surface map and the 3-D topographical image show very little carbonyl functionalization of the surface and equally very little variation across the surface (Figure 8). By contrast, sample PE-03 showed areas on the surface map where oxidation has occurred, alongside areas where very little surface chemical changes has occurred. The 3-D tomograph emphasizes this consistency with a relatively even topographical CI profile ranging from 0.2 to 0.5. PE-01 has an even more level CI tomograph ranging from 0.1 to 0.22 (Figure 8).

**Figure 9:**
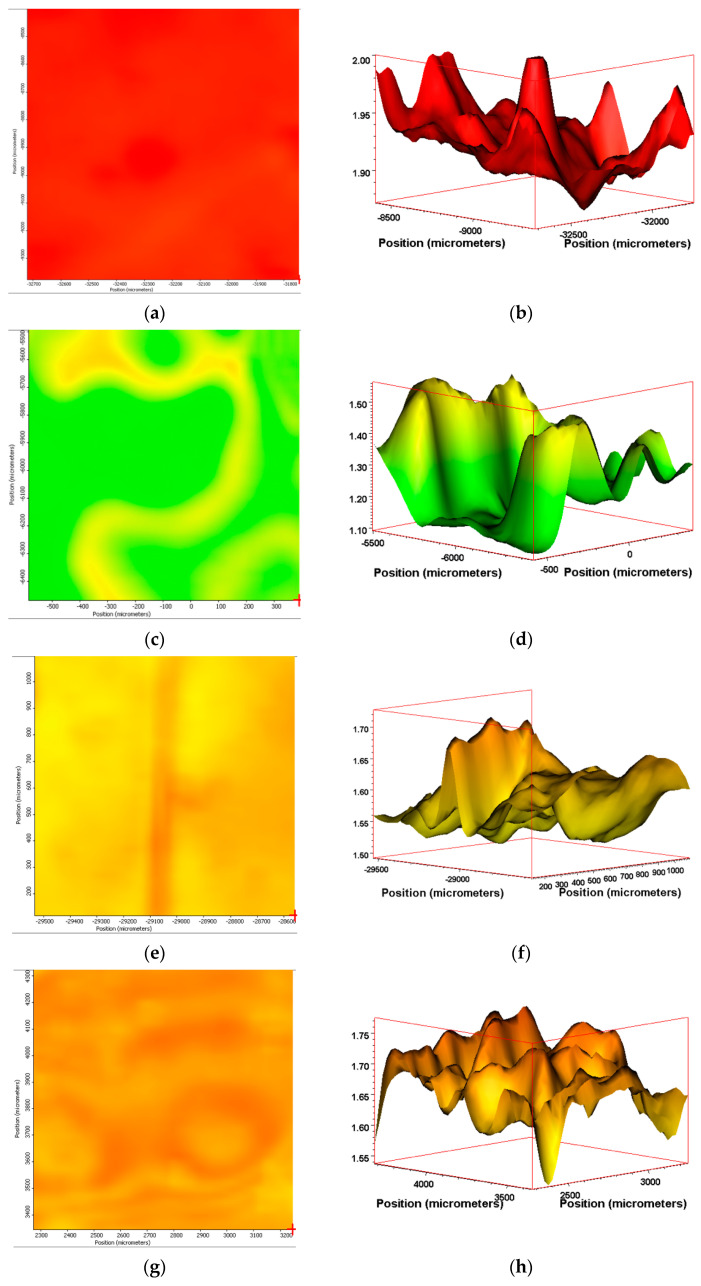

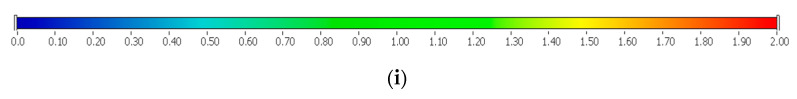
(**a**) IR ‘hotspot’ CI map and (**b**) corresponding 3-D CI tomograph of PE-02 after 14 days of temperate UV-accelerated weathering. (**c**) IR ‘hotspot’ CI map and (**d**) corresponding 3-D CI tomograph of PE-04 after 120 days of outdoor exposure in France. (**e**) IR ‘hotspot’ CI map and (**f**) corresponding 3-D CI tomograph of PE-05 after 90 days of outdoor exposure in Florida (summer). (**g**) IR ‘hotspot’ CI map and (**h**) corresponding 3-D CI tomograph of PE-06 after 90 days of outdoor exposure in Florida (winter). (**i**) Scale of ‘hotspot-map’ relative to CI values measured from IR spectra.

Sample PE-02 gives the highest calculated SAUB CI across the measured area of 1 mm^2^. The range of CI calculated is small, with a variance of 0.15, producing an effectively even ‘red’ surface image mapping when suing the ‘hotspot’ imagery (Figure 9a). When represented as a 3-D CI tomograph, the minor differences are evident in the CI ranging from 1.85 to 2.0 in the lowest troughs to the highest peaks, respectively (Figure 9b). Sample PE-04 has a larger divergence in the CI over the 1 mm^2^ surface area measured. The range is from 1.1 to 1.6, producing a green and yellow surface map with the equivalent ‘valley and peak’ in the 3-D tomographs (Figure 9c,d). The CI range of PE-04 (1.1–1.6) is approximately 3- to 4-fold higher as the range of CI measured for PE-03 (0.25–0.55) for an equivalent 1 mm^2^ area measured. PE-05 and PE-06 gives similar surface maps and 3-D CI tomographs post-exposure in Florida, despite being exposed at different times of the year. The range of CIs calculated is from 1.75 to 1.55 and 1.5 to 1.7 for PE-05 and PE-06, respectively (Figure 9e–h).

### 3.7. Drop Tests for Polyethylene Waxes

Polyethylene waxes are defined as polymerized ethylene which may be oxidized or copolymerized, but should have a melt (softening) point lower than 140 °C. Furthermore, ASTM D1986-14 [33] describes polyethylene waxes as not having a sharp solid–liquid phase change when heated and, therefore, they do not have a ‘definable’ melting point. Upon heating, these waxes gradually soften or become less viscous. A drop melting point is a more accurate description of the phase change and, whilst being arbitrary, can be measured reliably using the closely defined methodology in ASTM D127-19 [27] and ASTM D3954-15 [34]. The drop point test is used to determine the consistency and uniformity of the waxes in a reliable manner. The key physical property is the flow under gravity of the waxes at temperatures lower than 140 °C. Polyethylene waxes due to the lower molecular weights will flow at lower temperatures under gravity, whereas polymeric polyethylene materials would require an additional force or pressure to flow at temperatures below 140 °C. Using the methodology described in ASTM D127-19, we performed the drop tests on all the samples at the end of the associated weathering methodology (Table 5). The test is based around the visual observation of the test sample dropping as a molten wax from the tip of a thermometer at a specified temperature. If this phenomenon happens at a temperature below 140 °C, the sample is considered a polyethylene wax. If the test sample does not melt and subsequently does not drop under gravity, the sample is classified as polymeric polyethylene.

The drop tests show a clear correlation between the samples containing the biotransformation technology and those that do not. PE-01 and PE-03 still possess polymeric polyethylene physical properties of lack of flow under gravity at temperatures less than 140 °C. This observation is reinforced by the molecular weight analysis of these samples, which suggest a relatively small decrease in molecular weights during the weathering exposure. The Mw of PE-01 is approximately 110,671 Da, after temperate UV-accelerated weathering and the Mw of PE-03 is 36,198 Da after 120 days of outdoor exposure (Table 2 and Table 3). Both these values are higher than the 10,000 Da limit for the maximum molecular weight of a polyethylene wax as specified in ASTM D 1986-14 [33]. Therefore, when tested for the dropping point, both samples, post-weathering, exhibit the physical properties of a polymeric polyethylene sample. PE-02, -04, -05 and -06, however, show a significant decrease in their molecular weights during the weathering exposure, with all their Mw values less than 10,000 Da after their respective weathering. In addition, they also show a considerable increase in their CI of the surface of the samples (Figure 9). These changes in the chemical characteristics of the polyethylene structure within these samples changes their physical properties form those of a polymer to those of a polyethylene wax. These findings are confirmed by the visual observations of the drop testing, where the drop temperatures range from 114 to 118 °C.

The efficacy of the drop tests allowed for its further use to evaluate the samples PE-04 to PE-06 during their outdoor exposure. The drop testing was, therefore, further used at selected intervals during the outdoor exposure relating to the photographs taken during the exposure. The key intervals of interest were the images suggesting that the molecular weight reductions in the samples were resulting in physical disintegration of the test samples to waxes (See Section 4.2).

The results show that PE-03 starts to show signs of physical erosion at day 60 of outdoor exposure in France. Dropping test analysis of this sample, at this time point, suggests that material still contains the physical properties of polyethylene plastic, because the sample remains a solid up to 140 °C. In contrast, PE-04 and PE-05, also show signs of physical erosion at day 60 after outdoor exposure in France and Florida, respectively. The dropping points were determined to be 116 and 112 °C, respectively, suggesting that the sample displayed polyethylene wax properties. Finally, PE-05 showed an early onset of physical erosion at day 45. The dropping point was determined at 113 °C, suggesting that the sample had chemically converted to a wax before any physical erosion occurred.

## 4. Discussion

### 4.1. Comparison of Exposure Sites

Outdoor weathering in Florida is ubiquitously used in many industries from paints, plastics to automotive and aerospace to evaluate the effects of weather on materials and is used widely for the purpose of correlating laboratory weathering with outdoor exposure in the evaluation of plastic durability [1]. Due to the remoteness of the Florida exposure site, the average temperature experienced there was compared with the average temperatures for the same three months in the year 2020 for more-recognizable global cities (Table 6). A good correlation was found for the average temperatures observed in these cities with respect to the Florida exposure site, for Mumbai, Mombasa and Bangkok.

These testing sites and their correlation to similar climatic zones are of significance in the global awareness of plastic pollution. Recent studies have shown that the majority of terrestrial and marine sources of plastic pollution is grouped around areas that are situated in the Tropical Climatic Zone [6,7]. Furthermore, studies in the Mediterranean Sea have suggested that the Mediterranean Sea contains the highest quantity of microplastics compared to the other major marine environments [35,36]. This is presumed to be due to its geographical location and the subsequent number of different countries that surround its perimeter. For this region, laboratory methods that are comparable to ‘real-world’ environments are a key tool in understanding the formation of secondary microplastics, which after forming on land and are subsequently washed into the Mediterranean Sea. The data reported here demonstrate the utility and further potential of these UV-accelerated laboratory weathering cycles to predict the time and extent of secondary polyethylene microparticles due to exposure to environmental stimuli, even over a relative short period of time, such as 4 months.

**Figure 10:**
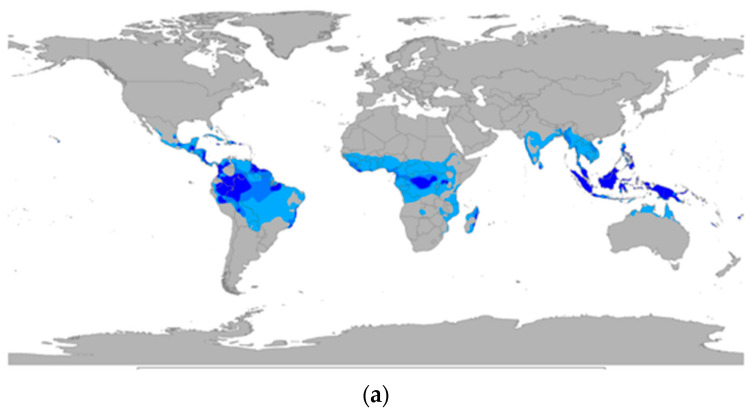

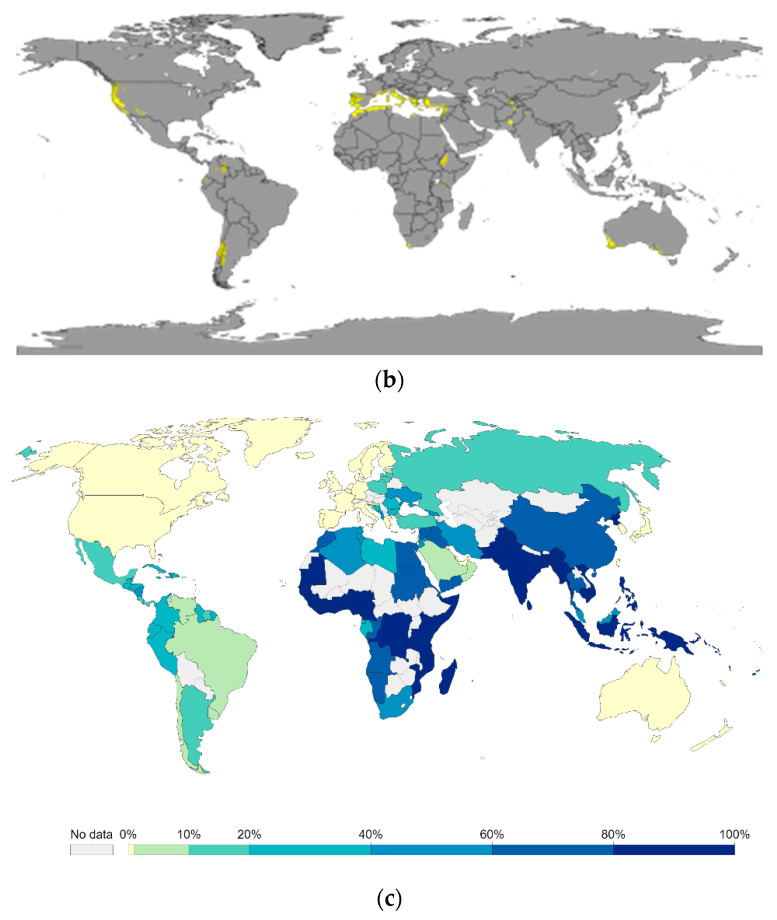
(**a**) Global image showing the Tropical Climate Zone, based upon the Koppen climate classification. (**b**) Global image showing the Mediterranean Climate Zone, based upon the Koppen climate classification. (**c**) Global image showing of known geographical areas of high % share of plastic pollution in 2010, based upon Jambeck et al. (2015).

More recent studies have shown that plastic pollution, originating from a terrestrial source is washing into the marine environment from over a 1000 different river sources. These sources are focused certain geographical regions more than others and seem to correlate with the initial studies performed by Jambeck et al. in 2015 [6] (Figure 10c). By stacking the climatic zones of correlation between the temperate laboratory UV-accelerated weathering cycles and the outdoor exposure alongside the global image of geographical areas of the highest percentage share of plastic pollution in 2010, the relevance of these experimental techniques is justified. The climate zones that the temperate UV-accelerated weathering cycles relate to are the same geographical zones where the plastic pollution is at its highest impact on the natural environment Thus, using these techniques will allow for a better understanding of how plastic pollution, through exposure the stimuli of the natural environment migrates, either as macro- or as micro-plastic particles from the land into the waterways, and eventually into the oceans.

Furthermore, the laboratory weathering techniques developed here allow for a rigorous evaluation of technologies designed to mitigate this pollution scenario. The biotransformation technology is designed to work, through a multi-faceted incorporation of chemical reactions, which cause both a decrease in the molecular weight of the polymer chains, as well as increased carbonyl functionalization of the low-molecular-weight and macromolecular waxes. The result of this technology and the aim of this study was to evaluate the carbonyl functionalized polyethylene waxes created through both temperate UV-accelerated weathering and outdoor exposure. To that end, PE-02, PE-04, PE-05 and PE-06 showed significant losses in the polyethylene chain structure under the scenarios of laboratory and outdoor exposure. The samples showed greater than 90% in both their Mw and Mz values within the first 20 to 40 days depending upon the testing environment. This agrees with a previous study in which a different mix of polyethylene resins made into a film was evaluated. (A predominantly LDPE to LLDPE mix [22], compared to the predominantly LLDPE to LDPE mix used in this work.) The suggested utility of this technology is an important performance criterion: firstly, because polyethylene films currently used in packaging can have a range of blended types of linear low- and low-density polyethylene, blended to give the processing and mechanical requirements desired by the producer, and secondly, because the differing resistances of each polyethylene resin type to environmental decomposition, would mean that the technology should be shown to be polyethylene resin agnostic in terms of its ability to reduce the molecular weight. LLDPE is known to be more environmentally resistant than LDPE [11,12] due to fewer side branches in the polyethylene chains. Demonstrating that the technology under evolution can reduce either type of polyethylene grade is crucial to the credibility of its performance [18]. Previous weathering cycles used in such cases have always drawn criticism that they have not shown any correlation to outdoor exposure [11] Thus, evaluating novel or innovative materials was difficult because of a lack of fundamental data demonstrating any correlation. Correlating the temperate weathering cycles to an equivalent outdoor exposure in this work has shown the utility of studying the effectiveness of technologies designed to combat plastic pollution in timeframes that are realistic to the problem.

### 4.2. Weathering Leading to Microparticles of Polyethylene or Polyethylene Waxes

One of the key environmental concerns when evaluating any type of plastic in the natural environment is the risk of the formation of microparticles from the plastic. Microplastics are currently defined as “all plastic particles with a size range between 100 microns and 5 mm”. The scientific community, especially polymer scientists, have riled at this non-scientific definition, as it only defines the size of the particles and not their physio-chemical definition, which can vary greatly depending upon the type of polymer under consideration. This criticism has recently been echoed by environment scientists, who state that the definition should be plastic type specific in order to be credible [37]. Furthermore, one of the key elements missing in the fight against plastic pollution, as stated by Hartmann et al., is better communication and collaboration between polymer and environmental scientists [37]. It is for this reason that in this and our previous work, we have explicitly brought molecular weight analysis to bear in the evaluation of the samples, so as to understand quantitively the changes within the polymer structure. Analysis in this work suggests that the PE films containing the biotransformation technology are no longer polymeric to the same degree as their original structure. They are, however, equally not macromolecules with a discrete chemical structure that can be elucidated by the types of analyses (NMR, GC–MS, etc.) used for the elucidation of fine chemicals.

In polymer science, polyethylene waxes are well-defined compounds with physical and chemical properties different to those of their polymeric polyethylene equivalents [38,39]. For example, their molecular weights are usually lower than 10,000 Da [33]. Consequently, they also have different physical properties, the most striking being their melt flow. Polyethylene waxes have a lower melt temperature and thus flow under an applied force (including gravity) at temperatures far lower than their polymeric polyethylene equivalents [34]. This desirable criterion, when used in applications such as lubrication, can be measured using a methodology codified in the drop point test of waxes, as defined in ASTM D127-19 [27]. We decided to employ this technique alongside the molecular weight analysis to present both the chemical and physical changes exemplified by microparticles of polyethylene compared to those of polyethylene waxes (Table 7).

The drop test demonstrates the flow under gravity of the sample at temperatures lower than 140 °C. Our aim was to combine the physical testing methodology of dropping point with chemical analysis in the form of the molecular weight determination for the various samples. In PE-03, the molecular weight reduction has not been sufficient to transform the polyethylene from a polymeric structure to an oligomeric polyethylene wax. Consequently, the temperate weathering technique, combined with this type of analysis, has also demonstrated how secondary microparticles of polyethylene are formed from primary macro-polyethylene sources. At day 60, the physical erosion caused deterioration of the polyethylene structure, resulting in micro fragments of polyethylene being produced after only 60 days of outdoor exposure in France. In contrast, the polyethylene samples containing the biotransformation technology, PE-04 to 06, produce polyethylene waxes after 120 days of outdoor exposure in France and Florida, respectively. Furthermore, this study has identified the point at which physical erosion occurs, as recorded by the visual observations: day 60 for PE-04 and PE-5; day 45 for PE-05 (Figure 10). By combining dropping point and molecular weight analysis, the waxes can be identified at the point at which physical erasure occurs. This suggests that for PE-04 and PE-06, from day 60 onwards, the sample can be considered a polyethylene wax rather than a polyethylene polymer. In the case of PE-05 exposed in the summer of Florida, this transformation has occurred from day 45 onwards. The data published here have shown the utility of combining ‘classical’ polyolefin analysis, high-temperature gel permeation chromatography, infrared spectroscopy and drop point testing, with a temperate UV-accelerated laboratory weathering cycle and natural outdoor exposure, to produce an in-depth study of the changes in the chemical and physical properties of polyethylene samples that can occur upon reaction with environmental stimuli.

## 5. Conclusions

This work sought to reinforce the utility of temperate laboratory UV-accelerated weathering cycles by correlating their exposure time to real-world geographical testing sites in Florida and France, representing two different climate zones. The study of standard polyethylene films using these weathering techniques has revealed the formation of secondary microparticles of polyethylene formed during the exposure time. This was corroborated by molecular weight and drop test analysis, which revealed the polyethylene chains had an average weight molecular weight of greater than 10,000 Da. By contrast, the samples containing the biotransformation technology suggested an induced change in physical and chemical state of the samples, from polymeric polyethylene to polyethylene waxes with a high level of functionalization, upon exposure to laboratory or outdoor weathering. The creation of these waxes was confirmed by a combination of molecular weight analysis, showing all had a weight-average molecular weight of less than 10,000 Da, a dropping point of below 140 °C, and an extensive level of carbonyl groups across the surface of the sample, as demonstrated by infrared spectroscopic mapping.

## Figures and Tables

**Table 1:** Sample names and descriptions of film compositions and exposure technique applied.

Sample Name	Description of Weathering Exposure
PE-01	Standard PE film weathered under temperate UV-accelerated laboratory conditions.
PE-02	Enhanced PE film containing the biotransformation technology weathered under temperate UV-accelerated laboratory conditions.
PE-03	Standard PE film weathered under outdoor exposure at Sanary Sur Mer, France between September and December 2020.
PE-04	Enhanced PE film containing the biotransformation technology weathered under outdoor exposure at Sanary Sur Mer, France between September and December 2020.
PE-05	Enhanced PE film containing the biotransformation technology weathered under outdoor exposure at Homestead, Florida between July and October 2020.
PE-06	Enhanced PE film containing the biotransformation technology weathered under outdoor exposure at Homestead, Florida between October 2019 and January 2020.

**Table 2:** Results of the PE-01 and PE-02 films tested under UV-accelerated laboratory conditions.

Total No. of Days of Exposure (Days/Hours)	Runtime Fraction	PE-01				PE-02			
		Mw (Da)	% Mw Loss	Mz (Da)	% Mz Loss	Mw (Da)	% Mw Loss	Mz (Da)	% Mz Loss
0 d	0	110,018	/	251,393	/	151,588	/	459,400	/
0.92 d (22 h)	0.067	109,470	28%	281,419	24%	24,746	78%	47,063	90%
3 d (72 h)	0.21	105,799	30%	241,274	34%	9815	91%	19,873	96%
6 d (144 h)	0.43	114,936	24%	311,659	15%	8099	93%	16,581	96%
7 d (168 h)	0.5	107,947	29%	254,525	31%	7301	93%	14,260	97%
10 d (240 h)	0.71	103,704	32%	230,252	37%	5397	95%	11,052	98%
12 d (288 h)	0.86	105,529	30%	246,260	33%	5663	95%	12,884	97%
14 d (336 h)	1	110,671	27%	279,801	24%	5397	95%	11,929	97%

**Table 3:** Results of the PE films under outdoor exposure at Sanary Sur Mer, France between September and December 2020.

Total No. of Days of Exposure (Days/Hours)	Runtime Fraction	PE-03				PE-04			
		Mw (Da)	% Mw Loss	Mz (Da)	% Mz Loss	Mw (Da)	% Mw Loss	Mz (Da)	% Mz Loss
0 d	0	110,018	/	251,393	/	151,588	0%	459,400	/
30 d (720 h)	0.25	81,032	26%	180,593	28%	19,494	87%	44,833	90%
60 d (1440 h)	0.5	79,746	28%	212,682	15%	16,779	89%	38,360	92%
90 (2160 h)	0.75	37,029	66%	87,500	65%	11,566	92%	26,857	94%
120 (2880 h)	1	36,198	67%	104,311	59%	9532	94%	25,834	94%

**Table 4:** Results of the PE films samples under outdoor exposure at Homestead, Florida between July and October 2020 (PE-05) and October 2019 and January 2020 (PE-06).

Total No. of Days of Exposure (Days/Hours)	Runtime Fraction	PE-05				PE-06					
		Mw	% Mw Loss	Mz	% Mz Loss	Time	Runtime Fraction	Mw	% Mw Loss	Mz	% Mz Loss
0 d	0	151,588	/	459,400	/	0 d	0	151,588	/	459,400	/
6 d (144 h)	0.078	90,062	41%	232,866	49%	12 d (288 h)	0.13	13,575	91%	32,942	93%
19 d (456 h)	0.21	17,068	89%	48,171	90%	25 d (600 h)	0.28	15,234	90%	57,343	88%
39 d (936 h)	0.43	8574	94%	19,450	96%	30 d (720 h)	0.33	8318	95%	18,668	96%
45 d (1080 h)	0.50	6241	96%	13,190	97%	42 d (1008 h)	0.47	6907	95%	16,550	96%
64 d (1536 h)	0.71	5529	97%	12,690	97%	60 d (1440 h)	0.67	7676	95%	16,912	96%
77 d (1848 h)	0.86	4850	97%	10,955	98%	72 d (1728 h)	0.80	4475	97%	10,325	98%
90 d (2160 h)	1	4694	97%	16,885	96%	90 d (2160 h)	1	3936	97%	9179	98%

**Table 5:** Drop testing as per ASTM D127-19 to determine if the sample could be classified as a polyethylene wax.

Sample	Weathering Protocol	Temperature of Dropping Point	Visual Observation at Drop Temperature (Optical Zoom 2×; Aspect Ratio 1:1; Images Cropped to Show Area of Thermometer Bulb 1 cm by 0.5 cm at Dropping Point)
PE-01	14 days Temperate UV-accelerated weathering	No drop	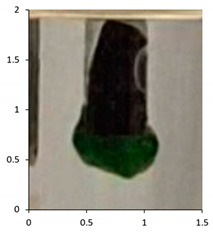
PE-02	14 days Temperate UV-accelerated weathering	114 °C	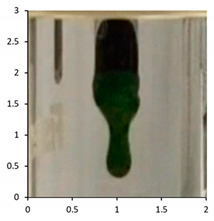
PE-03	120 days Outdoor exposure—France	No drop	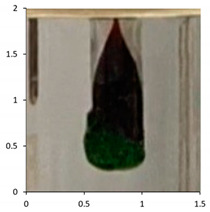
PE-04	120 days Outdoor exposure—France	114 °C	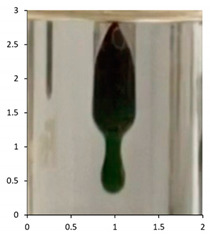
PE-05	90 days Outdoor exposure—Florida (Summer)	118 °C	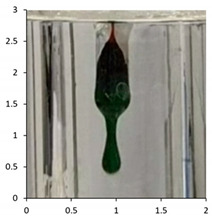
PE-06	90 days Outdoor exposure—Florida (Winter)	116 °C	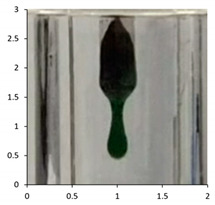

**Table 6:** Comparison between the average temperature in the outdoor weathering locations and cities around the world over the same period as the outdoor testing were conducted.

	Homestead, Florida ^[a]^	Sanary Sur Mer, France ^[b]^
Average daily temperature during testing exposure period	26 °C	15 °C
Average daily humidity during testing exposure period	84%	70%
Total rainfall during testing exposure period	323 mm	217 mm
Comparable global cities ^[c]^	Mumbai, India	Casablanca, Morocco
	Mombasa Kenya	Istanbul, Turkey
	Bangkok, India	Tel Aviv, Israel

^[a]^ Calculated from measurements performed by Q-Labs at the outdoor weathering site. ^[b]^ Calculated from measurements performed by Atlas at the outdoor weathering site. ^[c]^ Based on the Koppen climate classification.

**Table 7:** Dropping point of the test samples during outdoor exposure of (from top to bottom) PE-03, PE-04, PE-05 and PE-06. The sample bags have dimensions (height × width) of 30 cm × 25 cm. The magnification of the optical photographs was ×2.

PE-03	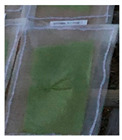	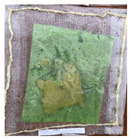	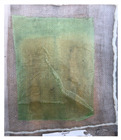
	Day 0 Dropping point—above 140	Day 60 Dropping point—above 140	Day 120 Dropping point—above 140
PE-04	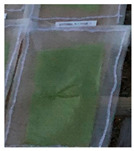	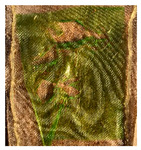	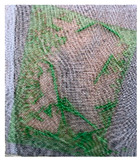
	Day 0 Dropping point—above 140	Day 60 Dropping point—116	Day 120 Dropping point—114
PE-05	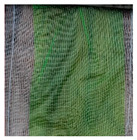	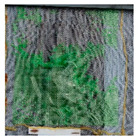	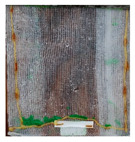
	Day 0 Dropping point—above 140	Day 45 Dropping point—above 113	Day 90 Dropping point—118
PE-06	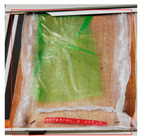	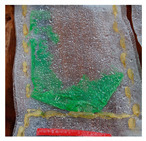	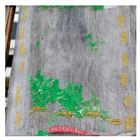
	Day 0 Dropping point—above 140	Day 60 Dropping point—112	Day 90 Dropping point—116

## Data Availability

Not applicable.

## Supplementary Materials

The following are available online at https://www.mdpi.com/article/10.3390/polym13142373/s1, Figure S1. Data showing temperature, levels of precipitation and relative humidity for South of France up to 91 days; Figure S2. Data showing temperature, levels of precipitation and relative humidity for Florida (Summer) up to 91 days; Figure S3. Data showing temperature, levels of precipitation and relative humidity for Florida (Winter) up to 91 days; Table S1. Average weather conditions over the exposure period; Table S2. Table showing the results of the PE films during the temperate UV-accelerated laboratory weathering; Table S3. Table showing the results of the PE films samples from Outdoor Weathering in France; Table S4. Table showing the results of the PE films samples from Outdoor Weathering in Florida Summer and Winter; Table S5. Table showing the drop point testing results of the PE films samples in triplicate with standard deviation; Figure S4. Atlas certificate for the 120-day outdoor exposure of PE-03 and PE-04 in France; Figure S5. Q-Lab certificate for the 90-day outdoor exposure of PE-05 in Florida; Figure S6. Q-Lab certificate for the 90-day outdoor exposure of PE-06 in Florida.
