# Supplementary material for: Temperate UV-Accelerated Weathering Cycle Combined with HT-GPC Analysis and Drop Point Testing for Determining the Environmental Instability of Polyethylene Films
Source: Polymers (Basel). 2021 Jul 20;13(14):2373. doi: 10.3390/polym13142373 (PMC8309575; doi:10.3390/polym13142373)
Supplement: Supplementary file 1 [file polymers-13-02373-s001.zip › polymers-1277133-supplementary.pdf]

polymers

MDPI

Supplementary Materials

Temperate UV-Accelerated Weathering Cycle Combined with HT-GPC Analysis and Drop Point Testing for Determining the Environmental Instability of Polyethylene Films

Celine Moreira, Richard Lloyd, Gavin Hill, Florence Huynh, Ana Trufasila, Faith Ly, Hasan Sawal and Christopher Wallis *

Polymateria Limited, i-Hub, Imperial College White City Campus, 84 Wood Lane, London, W12 0BZ, UK; cm@polymateria.com (C.M.); rl@polymateria.com (R.L.); gh@polymateria.com (G.H.); fh@polymateria.com (F.H.); at@polymateria.com (A.T.); fl@polymateria.com (F.L.); hs@polymateria.com (H.S.)
* Correspondence: cw@polymateria.com

S1. Weathering Data

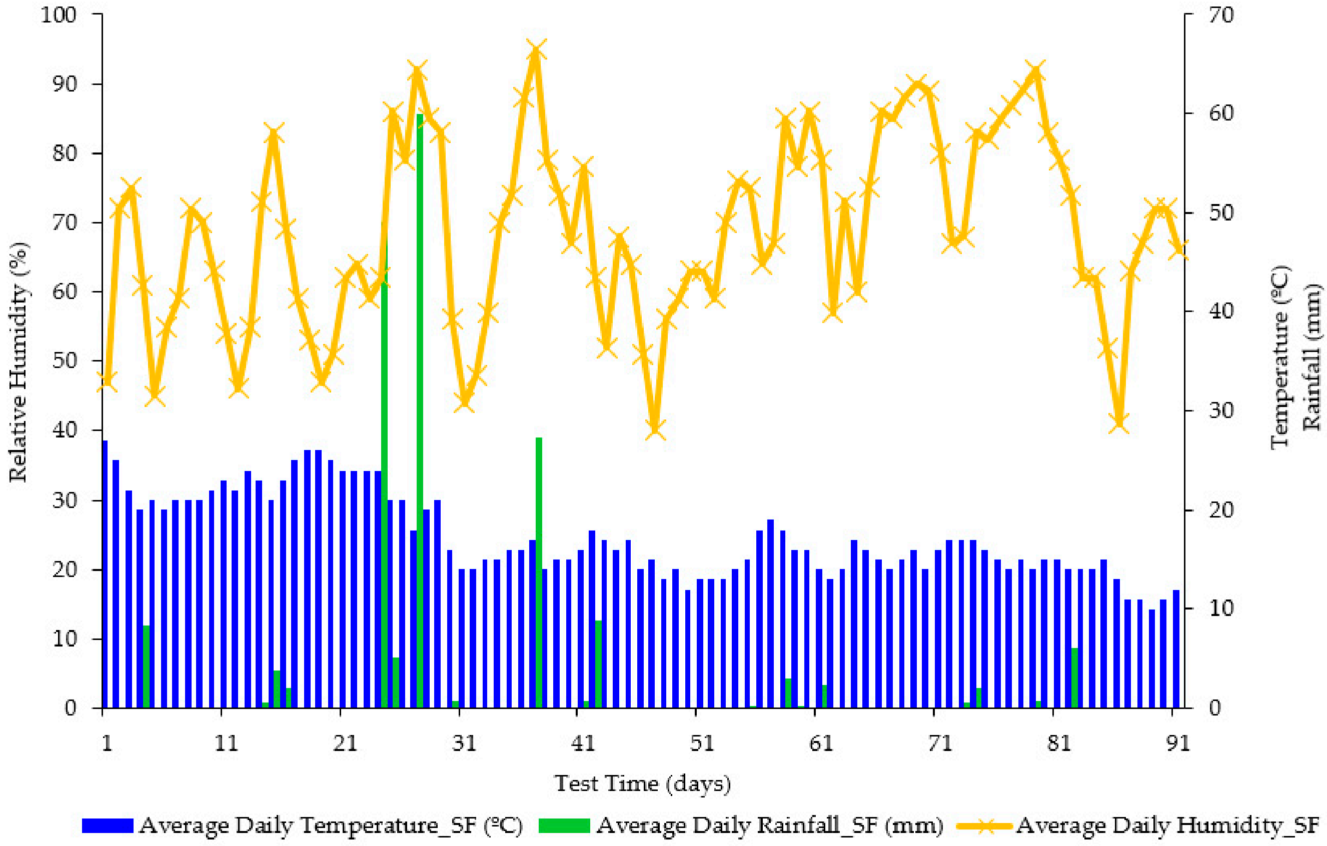

Figure S1. Data showing temperature, levels of precipitation and relative humidity for South of France up to 91 days.

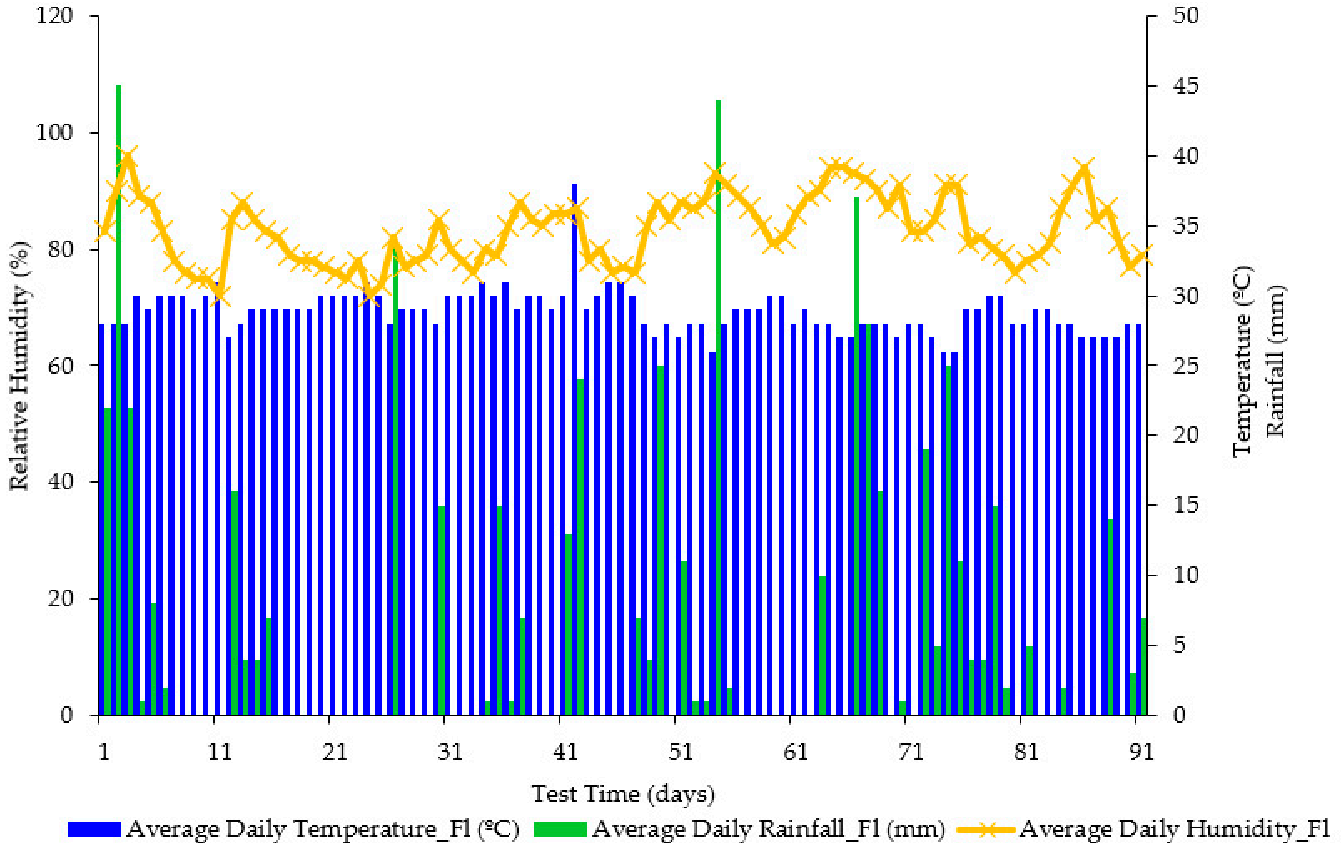

Figure S2. Data showing temperature, levels of precipitation and relative humidity for Florida (Summer) up to 91 days.

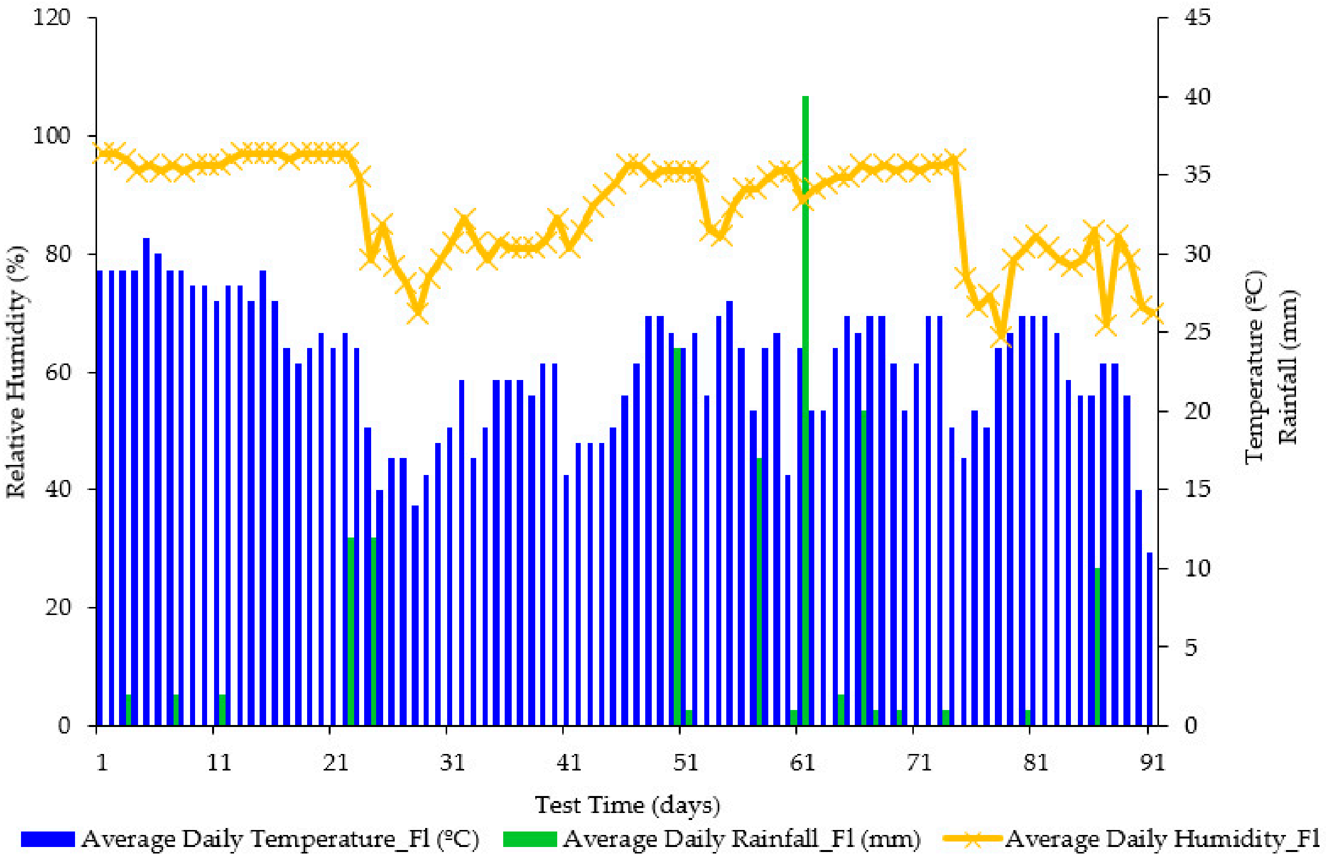

Figure S3. Data showing temperature, levels of precipitation and relative humidity for Florida (Winter) up to 91 days.

Table S1. Average weather conditions over the exposure period.

Expsoure Site	Calendar period of expsoure of sample	Average daily temperature during testing exposure period	Average daily humidity during testing exposure period	Total rainfall during testing exposure period
**Sanary Sur Mer, France**[b]	27th August – 25th Decemeber 2020	15.5°C	70.5%	217 mm
**Homestead, Florida**[a]	21st July – 19th October 2020	28.8°C	83.2%	498 mm
**Homestead, Florida**[a]	24th October 2019 – 22nd January 2020	22.9°C	87.8%	149 mm

S2. Moleculaer Weight Analysis

Table S2. Table showing the results of the PE films during the temeprate UV-accelerated laboratory weathering.

		PE-01					PE-02				
	Runtime Fraction	CI	Mn	Mw	Mw Loss (%)	Mz	CI	Mn	Mw	Mw Loss (%)	Mz
0	0	0.12	37,616	110,018	0	251,393	0.09	21,554	151,588	0	459,400
0.92	0.067	0.06	31,011	109,470	28%	281,419	0.15	8,571	24,746	78%	47,063
3	0.21	0.07	33,679	105,799	30%	241,274	0.75	3,266	9,815	91%	19,873
6	0.43	0.07	32,869	114,936	24%	311,659	0.91	3,107	8,099	93%	16,581
7	0.5	0.11	34,453	107,947	29%	254,525	0.69	2,936	7,301	93%	14,260
10	0.71	0.03	34,616	103,704	32%	230,252	1.39	1,979	5,397	95%	11,052
12	0.86	0.14	31,349	105,529	30%	246,260	1.19	2,062	5,663	95%	12,884
14	1	0.22	32,366	110,671	27%	279,801	1.60	1,956	5,397	95%	11,929

Table S3. Table showing the results of the PE films samples from Outdoor Weathering in France.

Time	Runtime Fraction	PE-03					PE-04				
		CI	Mn	Mw	Mw Loss (%)	Mz	CI	Mn	Mw	Mw Loss (%)	Mz
0	0	0.12	37,616	110,018	0%	251,393	0.09	21,554	151,588	0%	459,400
30	0.25	0.10	26,871	85,523	22%	193,906	0.32	5,175	19,494	87%	44,833
60	0.5	0.21	21,156	100,249	9%	262,538	0.44	3,451	16,779	89%	38,360
90	0.75	0.49	11,172	37,029	66%	87,500	0.52	3,319	11,566	92%	26,857
120	1	0.26	8,479	36,198	67%	104,311	0.62	2,119	9,532	93%	25,834

Table S4. Table showing the results of the PE films samples from Outdoor Weathering in Florida Summer and Winter.

Time	Runtime Fraction	PE-05					PE-06						
		CI	Mn	Mw	Mw Loss (%)	Mz	Time	Runtime Fraction	CI	Mn	Mw	Mw Loss (%)	Mz
0	0	0.09	21,554	151,588	0	459,400	0	0	0.09	151,588	21,554	0	459,400
6	0.078	0.09	26,454	90,062	41%	232,866	12	0.13	0.47	5,342	18,611	87%	43,886
19	0.21	0.37	4,412	17,068	89%	48,171	25	0.28	0.76	3,036	19,173	87%	63,950
39	0.43	1.03	2,487	8,574	94%	19,450	30	0.33	1.16	1,485	8,318	95%	18,668
45	0.50	1.07	1,676	6,241	96%	13,190	42	0.47	0.73	1,083	6,907	95%	16,550
64	0.71	1.36	1,485	5,529	97%	12,690	60	0.67	0.80	1,648	7,676	95%	16,912
77	0.86	1.39	1,306	4,850	97%	10,955	72	0.80	1.29	727	4,475	97%	10,325
90	1	1.53	1,020	4,694	97%	16,885	90	1	1.45	697	3,936	97%	9,179

Table S5. Table showing the drop point testing results of the PE films samples in triplicate with standard deviation.

Material	Time	Runtime Fraction	Dropping Point if below 140°C (°C)	Mean Dropping Point (°C)	Standard Deviation
Initial Blank	0	0	N/A	> 140 °C	N/A
			N/A		
			N/A		
Initial PLM	0	0	N/A	> 140 °C	N/A
			N/A		
			N/A		
PE-01	14	1	N/A	> 140 °C	N/A
			N/A		
			N/A		
PE-02	14	1	111	113	1.53
			113		
			114		
PE-03	60	0.5	N/A	> 140 °C	N/A
			N/A		
			N/A		
PE-03	120	1	N/A	> 140 °C	N/A
			N/A		
			N/A		
PE-04	60	0.5	116	114	1.53
			114		
			113		
PE-04	120	1	114	114	0.58
			115		
			114		
PE-05	45	0.5	113	112	1.53
			112		
			110		
PE-05	90	1	118	113	4.73
			111		
			109		
PE-06	45	0.5	115	113	1.53
			113		
			112		
PE-06	90	1	116	112	3.79
			110		
			109		

S3. Outdoor exposure Testing Certificates

ATLAS
MATERIAL TESTING SOLUTIONS

AMETEK
MEASUREMENT & CALIBRATION TECHNOLOGIES

Atlas Material Testing Technology B.V.
890 Chemin les Hautes du Lancon
83110 Sanary sur Mer
France
rachida.hajaji@ametek.com
Phone: +33 130 68 89 08
Fax: +33 1 30 68 89 99
R.C. Meaux B 329 572 903

Confidential

POLYMATERIA LIMITED
Mrs Celine Moreira
80 Wood Lane
W120BZ London
UK

Sanary, January 04, 2021

TEST REPORT
INSPECTION DATA AT 120 Days

Purchase Order Number: N/A; Release Number: N/A
Client Code: UKPOLCM

Test Number: **SS10897**

Report Number: 4
Test Type: INLAND WEATHERING
Test Location: Sanary, France
Specimens Inspected: 32
Description: 104 samples received ok

Exposure Type: Exposure testing is performed in Sanary, France in accordance with ISO877-1 "Plastics - Method of exposure to solar radiation - General Guidance" at a tilt angle(s) of 45° from the horizontal facing south. The specimens are mounted backed on 3mm exterior grade plywood on a 1643 x 3586 mm aluminum exposure rack, with grass groundcover, and the coded side facing the sun.

Observations, Deviations and Waivers
Notes contained in relevant documents are an integral part of a test, and shall be included by the client in discussions, correspondence, and presentation of test results to a third party. This Test Report represents only one part of the test documentation. Interim reports and other test documentation may have been submitted prior to this date. Test results reported are pertinent only to the items tested and are not relevant to other specimens of the same type, or in the same lot, which are not being tested. Test Reports, and/or other pertinent test documentation, shall not be reproduced, except in full, without the written approval of Atlas Weathering Services Group and so certified by the client.

ACCREDITED ISO/IEC 17025
717.07

Page 1 of 3

Figure S4. Atlas certificate for the 120-day outdoor exposure of PE-03 and PE-04 in France.

Q-LAB

Q-Lab Weathering Research Service
1005 S.W. 18 Avenue
P. O. Box 349490
Homestead, FL 33034

TEST CERTIFICATE

Natural Weathering

**Test Program Number:**	*PLL-4-TP-1*
**Company:**	*Polymateria Limited*
**Address:**	*Imperial College London I-HUB White City Campus 80 Wood Lane*<br>*London W12 0BZ,*<br>*UNITED KINGDOM*
**Attention:**	*Ms. Celine Moreira*
**Your Reference:**	*Project Plan 2*
**No. Of Specimens:**	*68*
**Specimen Identification:**	*See following page.*
**Test Method:**	*ASTM G7 2013*
**Deviations:**	*None*
**Exposure Date:**	*July 21, 2020*
**Completion Date:**	*October 19, 2020*
**Exposure Duration:**	*2 Months 28 Days*
**Exposure Type:**	*Direct Weathering- Florida*<br>*45° South*<br>*Backed*
**Radiation Exposure:**	*Total = 1,449.89 MJ/m2 TUVR = 81.53 MJ/m2*

By:

Rosie Rosario
Test Set Up Supervisor

Approved By:

Michael Crewdson
General Manager

Page 1 of 2

Figure S5. Q-Lab certificate for the 90-day outdoor exposure of PE-05 in Florida.

Q-LAB

Q-Lab Weathering Research Service
1005 S.W. 18 Avenue
P. O. Box 349490
Homestead, FL 33034

TEST CERTIFICATE

Natural Weathering

**Test Program Number:**	PLL-1-TP-1
**Company:**	Polymateria Limited
**Address:**	Imperial College London I-HUB White City Campus 80 Wood Lane<br>London W12 0BZ,<br>UNITED KINGDOM
**Attention:**	Ms. Celine Moreira
**Your Reference:**	
**No. Of Specimens:**	10
**Specimen Identification:**	See following page.
**Test Method:**	ASTM G7 2013
**Deviations:**	None
**Exposure Date:**	October 24, 2019
**Completion Date:**	January 22, 2020
**Exposure Duration:**	2 Months 29 Days
**Exposure Type:**	Direct Weathering- Florida<br>45° South<br>Backed
**Radiation Exposure:**	Total = 1,632.64 MJ/m2 TUVR = 69.52 MJ/m2

By: Rosie Rosario
Test Set Up Supervisor

Approved By: Michael Crewdson
General Manager

Figure S6. Q-Lab certificate for the 90-day outdoor exposure of PE-06 in Florida.
